# Intestinal malrotation with suspected cow’s milk allergy: a case report

**DOI:** 10.1186/1756-0500-5-481

**Published:** 2012-09-03

**Authors:** Takuma Matsuki, Akimune Kaga, Susumu Kanda, Yutaka Suzuki, Muneyuki Tanabu, Naoya Sawa

**Affiliations:** 1 Department of Pediatrics, Hachinohe City Hospital, 1 Bisyamontaira, Hachinohe, Aomori, 031-8555, Japan; 2 Department of Pediatrics, Tohoku University School of Medicine 1-1, Seiryo-machi, Aoba-ku, Sendai, Miyagi, 980-8574, Japan; 3 Department of Pediatric Surgery, Hachinohe City Hospital, 1 Bisyamontaira, Hachinohe, Aomori, 031-8555, Japan

**Keywords:** Allergen-specific lymphocyte stimulation test, Cow’s milk allergy, Food challenge test, Infant, Intestinal malrotation

## Abstract

**Background:**

Intestinal malrotation is an incomplete rotation of the intestine. Failure to rotate leads to abnormalities in intestinal positioning and attachment that leave obstructing bands across the duodenum and a narrow pedicle for the midgut loop, thus making it susceptible to volvulus. One of the important differential diagnoses for malrotation is an allergy to cow’s milk. Several studies have described infants with surgical gastrointestinal diseases and cow’s milk allergy. However, to our knowledge, no study has reported infants with intestinal malrotation who have been symptomatic before surgery was performed and have been examined by allergen-specific lymphocyte stimulation test and food challenge tests with long-term follow-up.

**Case presentation:**

The patient was a Japanese male born at 39 weeks of gestation. He was breast-fed and received commercial cow’s milk supplementation starting the day of birth and was admitted to our hospital at 6 days of age due to bilious vomiting. Plain abdominal radiography showed a paucity of gas in the distal bowel. Because we demonstrated malpositioning of the intestine by barium enema, we repositioned the bowel in a normal position by laparotomy. The patient was re-started on only breast milk 2 days post surgery because we suspected the presence of a cow’s milk allergy, and the results of an allergen-specific lymphocyte stimulation test showed a marked increase in lymphocyte response to kappa-casein. At 5 months of age, the patient was subjected to a cow’s milk challenge test. After the patient began feeding on cow’s milk, he had no symptoms and his laboratory investigations showed no abnormality. In addition, because the patient showed good weight gain and no symptoms with increased cow’s milk intake after discharge, we concluded that the present case was not the result of a cow’s milk allergy. At 1 year, the patient showed favorable growth and development, and serum allergy investigations revealed no reaction to cow’s milk.

**Conclusion:**

When physicians encounter infants with surgical gastrointestinal disease, including intestinal malrotation, they should consider cow’s milk allergy as a differential diagnosis or complication and should utilize food challenge tests for a definitive diagnosis.

## Background

Intestinal malrotation (IM) is the incomplete rotation of the intestine during fetal development [1]. Failure to rotate leads to abnormalities in intestinal positioning and attachment that may leave obstructing bands across the duodenum and a narrow pedicle for the midgut loop, thus rendering it susceptible to volvulus. Infants affected with IM often present during the first week of life with bilious emesis. The upper gastrointestinal series is the imaging test of choice and the gold standard in the evaluation and diagnosis of malrotation and volvulus [1]. A barium enema can also demonstrate malpositioning of the cecum [1]. If a volvulus is present, surgery is performed immediately because gastric obstruction is an acute emergency.

One of the important differential diagnoses for malrotation is cow’s milk allergy (CMA), which has been defined as an adverse reaction occurring after the ingestion of cow’s milk as a result of an immunologic hypersensitivity to milk protein [2]. Adverse reactions may have a wide range of clinical manifestations [2-4]. The diagnosis of CMA is difficult, although the allergen-specific lymphocyte stimulation test (ALST) [4,5], particularly the response to kappa-casein, is a useful diagnostic test [5]. The definitive diagnosis is made by a food challenge test [2-4].

Several studies have compared infants with surgical gastrointestinal disease and CMA [4,6,7], and only one English-language report has described infants with IM combined with CMA [8]. However, to our knowledge, no study has reported infants with IM who have been symptomatic before surgery was performed and have been examined by ALST and food challenge tests with long-term follow-up. In this article, we report a case of IM and suspected CMA.

## Case presentation

**Figure 1:**
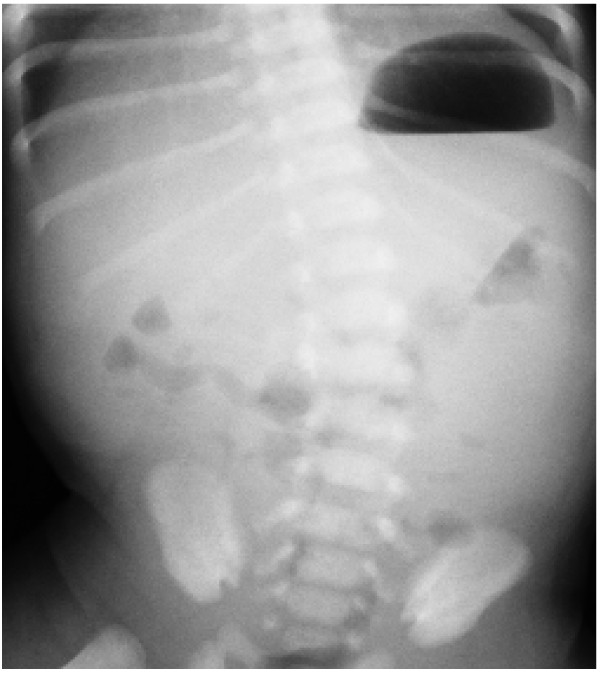
A plain abdominal radiograph shows a paucity of gas in the distal bowel.

**Figure 2:**
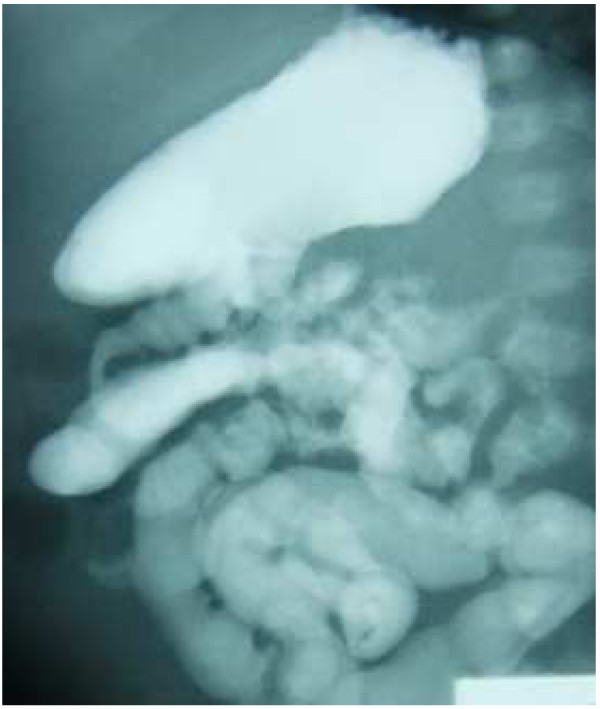
An upper gastrointestinal series shows normal positions of the duodenum and jejunum.

The patient was a Japanese male born at 39 weeks of gestation. He had no significant pre- or peri-natal history and was born after an uncomplicated delivery with Apgar scores of 9 at 1 minute and 10 at 5 minutes. On the day of birth, breast-feeding supplemented with commercial cow’s milk was started. The patient was admitted to our hospital at 6 days of age due to bilious vomiting. At the time of admission, he was in poor condition but had no abdominal distention. Laboratory investigations showed a total white cell count of 12,500/mm^3^ with 2% eosinophils, 14.9 g/dL hemoglobin, 245,000 platelets/mm^3^ and 0.17 mg/dL CRP with no eosinophilia in the stool, and serum allergy investigations revealed no remarkable elevation of non-specific IgE (1 IU/mL) and cow’s milk-spcific IgE (<0.34 IU/mL). Plain abdominal radiography showed a paucity of gas in the distal bowel (Figure 1). An upper gastrointestinal series showed that the duodenum and jejunum were normally positioned (Figure 2). The patient’s enteral feeding was stopped, he was administered antibiotics, and his clinical course was observed.

**Figure 3:**
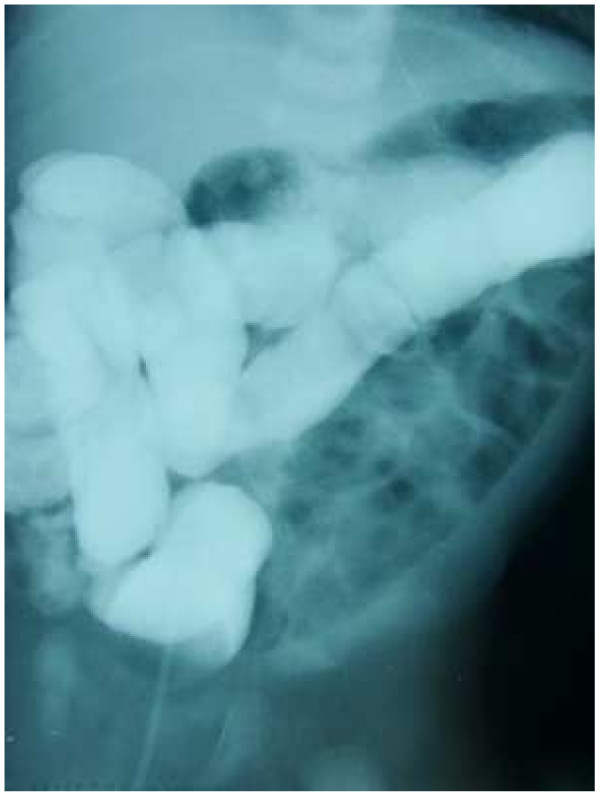
A barium enema shows malpositioning of the intestine.

On the following day, specks of blood mixed with mucus were visible in the stool, and a barium enema revealed malpositioning of the intestine (Figure 3). A laparotomy was performed, and it was observed that the ascending colon and descending colon were not attached and moved freely, the ligament of Treitz was in the normal position, and no intestinal ischemia was observed. We therefore returned the bowel to a normal position.

Because the patient’s lymphocyte response to kappa-casein was markedly increased (10,612 cpm; stimulation index 10), we suspected that he had a CMA, and the patient was re-started on breast milk alone 2 days post-surgery. Thereafter, the patient showed symptom-free normal progress with good weight gain.

At 5 months of age, the patient was admitted to our hospital to undergo a cow’s milk challenge with parental consent before eating anything other than breast milk. After the patient was started on cow’s milk feeds, he remained asymptomatic, and laboratory analyses showed no elevation of white cell count or CRP, and no eosinophilia in the blood or stool. In addition, the patient showed good weight gain and no symptoms with increased cow’s milk intake after discharge. Therefore, we concluded that this case was not the result of a CMA.

At 1 year of age, the patient showed favorable growth and development, and serum allergy investigations revealed no remarkable elevation of nonspecific IgE (74 IU/mL) and no reaction to cow’s milk.

## Discussion

Several studies have reported an association between infants with surgical gastrointestinal disease and CMA [4,6,7]. Only one English-language report has described children with both IM and CMA [8]. However, to our knowledge, there have been no reports of infants with IM who were examined by ALST and food challenge tests with a long-term follow-up.

In recent years, it has been suggested that the development of CMA after gastrointestinal surgery in newborn infants is due to an immature intestinal mucosal barrier and related immune function [4,6]. In addition, congenital abnormalities of the intestinal mucosa, general conditional changes and local damage to the intestine by invasive surgery and pre- or post-surgical under nutrition may act synergistically to aid CMA development [4,6]. In the present case, we concluded that an upper gastrointestinal series showed normal positioning at the time of admission because the ligament of Treitz was in the normal position and the ascending colon and descending colon were not attached and moved freely. The patient was diagnosed with IM because a barium enema and laparotomy showed malpositioning of the intestine. We speculated that the intestinal tract of the patient was blocked by a volvuls.

A previous report described malrotation in children with symptoms of gastrointestinal allergy and psychosomatic abdominal pain [8]. Three children with malrotation who were 4.5, 5 and 9 years old at the time of surgery were presented. Their preliminary diagnoses were gastrointestinal allergy irritable colon and psychosomatic abdominal pain. They were treated on an outpatient basis under these diagnoses for more than two years before their malrotations were discovered. They have been symptom free since their surgeries were performed [8]. They were not examined by ALST and food challenge testing. The present case differed from previous reports with regard to the age of the patient and the performance of ALST and a food challenge test.

The ALST is a very useful laboratory test for the diagnosis of CMA [4,5], and the positivity rate is 88% [5]. Kappa-casein has the most potent capacity to activate lymphocytes [5]. In this case, although kappa-casein induced a high lymphocyte proliferative response, we concluded that the patient did not have a CMA, because the food challenge test was negative. We speculated that kappa-casein may be highly activating when intestines suffer a strong insult such as voluvuls. Further studies are needed to clarify why various patterns were observed.

Adverse reactions to cow’s milk are frequent in the first year of life [9]. Half of children tolerate cow’s milk by age 1 [9]. A challenge test with the causal food is indispensable in the diagnosis of food allergy, but is not realistic for all cases in newborn babies [10]. Few institutions in Japan have reported that they routinely perform a challenge test with cow’s milk for all cases of CMA [10]. Inappropriate dietary restriction independent of adequate medical and dietary supervision can cause morbidity in the infant or mother (or both), through the inadequate intake of dietary components, especially calcium [3].

In the future, through the evaluation of more cases with sufficient laboratory data, a more complete classification should be possible. This classification can be applied to a study of the prognosis of this rare condition.

## Conclusion

We report a case of a child with IM and suspected CMA. If physicians encounter infants with surgical gastrointestinal disease including IM, they need to consider CMA in the differential diagnosis or as a complicating factor. While the ALST is a useful laboratory test for the diagnosis of CMA, it may be highly reactive when the intestine is strongly inflamed. The physician should perform food challenge tests for a definitive diagnosis.

## Consent

Written informed consent was obtained from the patient’s guardian for publication of this case report and accompanying images. A copy of the written consent is available for review by the Editor-in-Chief of this journal.

## Abbreviations

ALST: Allergen-specific lymphocyte stimulation test; CMA: Cow’s milk allergy; IM: Intestinal malrotation.

## Competing interests

The authors declare that they have no competing interests.

## Authors’ contributions

TM, AK, SK, YS MT and NS treated the patient and in doing so acquired the case data; they were also involved with drafting of the manuscript. All authors read and approved the final manuscript.
